# Dichlorvos exposure results in large scale disruption of energy metabolism in the liver of the zebrafish, *Danio rerio*

**DOI:** 10.1186/s12864-015-1941-2

**Published:** 2015-10-24

**Authors:** Tri M. Bui-Nguyen, Christine E. Baer, John A. Lewis, Dongren Yang, Pamela J. Lein, David A. Jackson

**Affiliations:** ORISE Postdoctoral Fellow, Fort Detrick, MD 21702 USA; Excet, Inc., Springfield, VA 22151 USA; US Army Center for Environmental Health Research, Fort Detrick, MD 21702 USA; Molecular Biosciences, School of Veterinary Medicine, University of California Davis, Davis, CA 95616 USA; Current address: US Food and Drug Administration, Silver Spring, MD 20993 USA

**Keywords:** Zebrafish, Dichlorvos, Microarray, Organophosphorus, Fasting

## Abstract

**Background:**

Exposure to dichlorvos (DDVP), an organophosphorus pesticide, is known to result in neurotoxicity as well as other metabolic perturbations. However, the molecular causes of DDVP toxicity are poorly understood, especially in cells other than neurons and muscle cells. To obtain a better understanding of the process of non-neuronal DDVP toxicity, we exposed zebrafish to different concentrations of DDVP, and investigated the resulting changes in liver histology and gene transcription.

**Results:**

Functional enrichment analysis of genes affected by DDVP exposure identified a number of processes involved in energy utilization and stress response in the liver. The abundance of transcripts for proteins involved in glucose metabolism was profoundly affected, suggesting that carbon flux might be diverted toward the pentose phosphate pathway to compensate for an elevated demand for energy and reducing equivalents for detoxification. Strikingly, many transcripts for molecules involved in β-oxidation and fatty acid synthesis were down-regulated. We found increases in message levels for molecules involved in reactive oxygen species responses as well as ubiquitination, proteasomal degradation, and autophagy.

To ensure that the effects of DDVP on energy metabolism were not simply a consequence of poor feeding because of neuromuscular impairment, we fasted fish for 29 or 50 h and analyzed liver gene expression in them. The patterns of gene expression for energy metabolism in fasted and DDVP-exposed fish were markedly different.

**Conclusion:**

We observed coordinated changes in the expression of a large number of genes involved in energy metabolism and responses to oxidative stress. These results argue that an appreciable part of the effect of DDVP is on energy metabolism and is regulated at the message level. Although we observed some evidence of neuromuscular impairment in exposed fish that may have resulted in reduced feeding, the alterations in gene expression in exposed fish cannot readily be explained by nutrient deprivation.

**Electronic supplementary material:**

The online version of this article (doi:10.1186/s12864-015-1941-2) contains supplementary material, which is available to authorized users.

## Background

Hundreds of pesticides are used to enhance food production and control disease vectors worldwide. Dichlorvos (DDVP), an organophosphorus pesticide used for indoor insect and livestock parasite control, is among the most common commercially available pesticides. There are, however, significant concerns over its acute and chronic toxicity [1–3], especially because dichlorvos is relatively stable in water, soil, and air [4], with a half life ranging from two (air) to several days or weeks (water and soil).

DDVP reversibly inhibits acetyl cholinesterase (AChE) activity leading to an accumulation of acetylcholine and increased cholinergic activity in the central and peripheral nervous system, and at neuromuscular junctions [3, 4]. Exposure does not appear to result in genotoxic or teratogenic effects, but it may result in pancreatic adenoma, mononuclear leukemia, mammary gland carcinoma, fibroadenoma, and adenoma [5, 6]. Following long term chronic exposure to DDVP, rats displayed hepatocellular vacuoles and cell swelling [7]. In neuronal tissue of rats, DDVP exposure alters carbohydrate homeostasis [8, 9] and induces damage that is likely due to oxidative stress [10], and the testes of chronically DDVP–exposed rats display mitochondrial swelling, necrosis, and edema in the seminiferous tubules [11].

There is substantially less information on the effects of dichlorvos on fish, but the chemical is known to cause oxidative stress, liver pathology, cholinesterase inhibition and neurobehavioral abnormalities, as well as injuries to the skin and gills in various species [12]. The primary routes of exposure are presumed to be through the gills and skin like most pesticides in fish [13].

The liver is a primary site of DDVP metabolism [14], where it is converted to desmethyl-dichlorvos, dimethyl phosphate, and dichloroacetaldehyde by glutathione-dependent and aryl esterase pathways [4, 15–17]. While the metabolism of DDVP was initially described decades ago [16], the cause of its toxicity in non-neural target organs such as the liver or kidney remains unclear.

In an effort to elucidate the mechanisms of DDVP toxicity, we explored changes in gene expression in the livers of zebrafish exposed to DDVP. Toxicity testing in zebrafish can potentially provide a number of practical advantages over studies in rodents or other mammals. In particular, since zebrafish are a non-mammalian species, using them can contribute to satisfying both the replacement and reduction principles in animal research by reducing the number of mammals required for definitive studies [18]. Moreover, zebrafish culture is convenient and inexpensive, and the fish provide a system to model toxicity in mammals [19]. Additionally, the use of flow-through aquatic exposure system can provide a constant easily controlled exposure environment (see Methods). With phenotypes resembling human diseases, similarities in anatomy, biochemistry, and genetics [20], the zebrafish is an attractive model system for many biomedical questions. Results from zebrafish studies are generally complementary to those from other animal model systems, and demonstrate both physiological and biochemical similarities to xenobiotic metabolism and adaptive responses to toxicants in mammals [21–23]. Indeed, the zebrafish model has been used extensively to assess organ toxicity in the liver, pancreas, and intestine [22, 24].

Although a number of prior studies have investigated the effects of dichlorvos on particular aspects of liver metabolism, they focused on particular metabolites or enzymes [25, 26]. To obtain a broader understanding of the effects of dichlorvos on liver metabolism, we performed a genome-wide analysis of gene expression in the liver of zebrafish exposed to DDVP and identified biological responses to DDVP using a functional analysis of differentially expressed genes. The affected processes are similar to those reported in mammals [27, 28]. Our global transcriptomic analysis revealed functionally related changes in the expression of a large number of genes involved in carbohydrate and lipid metabolism, responses to reactive oxygen insult, and autophagy in contrast to the mammalian studies, which focused only on a small number of metabolites or enzymatic activities. To ensure that these changes were not simply the result of poor feeding and nutrient deprivation in fish with impaired neuromuscular function, we compared the patterns of gene expression between fasted and DDVP-exposed fish. The pattern of gene expression in the DDVP exposed fish was distinct from that in the fasted fish. The functional relationships among the genes differentially expressed in response to DDVP imply that exposure results in oxidative stress and stimulates a coordinated adaptive response, at least in part at the transcriptional level, to mitigate a redox imbalance.

## Results and discussion

To investigate the effects of DDVP exposure on the liver, we exposed male zebrafish to three concentrations of DDVP for 24 h, harvested the livers of one set of fish (*n* = 20/condition) for whole genome transcript analysis, and fixed another set of fish for histological evaluation (*n* = 5/condition). We determined the target concentrations of DDVP for exposure based on 96 h range finding experiments for the no- observable- effect level (6 mg/L) as well as for DDVP concentrations that resulted in the death of 20 % (19 mg/L) or 60 % (32 mg/L) of the fish (Additional file 1). The measured concentrations in the exposure experiment were (6, 16, and 30 mg/L [27, 72, and 135 μM])) which we refer to as low, mid, and high. To identify gene expression responses characteristic of intoxication but preceding organ failure and death, we exposed the fish to DDVP for only 24 h in the definitive experiment.

### Cholinesterase activity

**Fig. 1:**
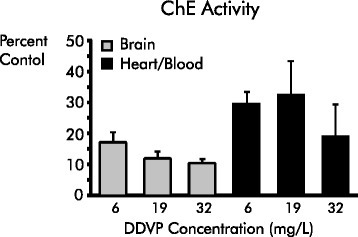
DDVP exposure reduces cholinesterase activity in brain and heart. Hearts and brains from four control and four exposed fish were assayed for cholinesterase activity by the Ellman method. Data are presented as percentages of unexposed control. All exposed conditions differed from control at the *p* ≤ 0.05 level using the Kruskal-Wallis test. Error bars represent the standard error of the mean (SEM)

We confirmed the effectiveness of the DDVP exposure by measuring cholinesterase (ChE) activity in extracts from brain and heart tissue using the Ellman method; heart ChE activity is considered to result chiefly from the presence of residual blood in the organ [29]. As shown in Fig. 1, ChE activity was inhibited up to 80 % in the heart tissue of exposed zebrafish when compared to control and 90 % in brain ChE activity (*p* < 0.05). Previous studies have shown that DDVP inhibits ChE activity in the fish *Tilapia. mossambica* [30] and in gilthead sea bream (*Sparus aurata*) brain and muscle tissues [31].

### Histopathological examination

**Fig. 2:**
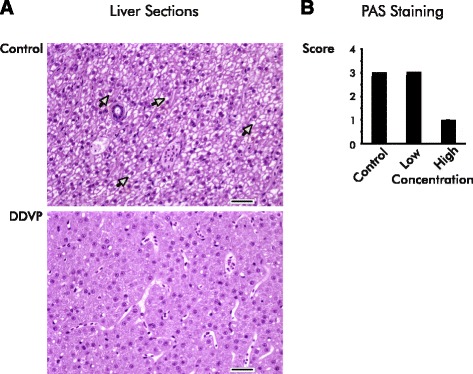
Glycogen vacuolation is reduced in the livers of DDVP–exposed fish. **a** The liver of exposed fish shows decreased hepatocellular vacuolation compared with control animals (*yellow arrows*). Representative images of transversely sectioned hematoxylin and eosin stained livers from a control and a unexposed fish (high concentration) are shown. Bars are 25 μm. **b** Periodic Acid-Schiff (PAS) staining reveals reduced levels of glycogen in the livers of exposed fish. Liver sections from controls and fish exposed to high and low concentrations of DDVP were semi-quantitatively scored for PAS-positive glycogen staining (0-4+, low to high staining intensity). All unexposed fish were scored “3 + .” Scores for the low concentration-exposed fish ranged from 2 + −4+ (mean = 3+), and all the fish exposed to the high concentration were scored “0–1+”. Exposed fish differed from control at *p* ≤ 0.05 level using the Kruskal-Wallis test. The high concentration exposure was significantly different from control in *post hoc* testing with the Wilcoxon ranks test (*p* ≤ 0.05)

While organophosphates are usually thought of as affecting nerve and muscle, semi-quantitative histopathological analysis showed that DDVP exposure affected multiple organs in the zebrafish. In addition to muscle, effects on testis, pancreas, kidney, and liver were observed (Table 1 and Fig. 2; see Additional file 2 for photomicrographs of representative tissue sections from skeletal muscle, testis, pancreas, and kidney and additional description).

**Table 1:** DDVP exposure results in injury to non-neural tissues

Exposure					
Observation		Control	Low	Mid	High
• Myodegeneration	Mean Score	0	0	0.4	2.4^†^
	Prevalence	0/4	0/5	2/5	5/5
• Testis degeneration	Mean Score	0.2	0.4	2.2^†^	2.8^†^
	Prevalence	1/4	2/5	5/5	5/5
• Pancreas zymogen granule depletion	Mean Score	0	0	2.2^†^	2.4^†^
	Prevalence	0/4	0/5	4/5	5/5
Renal congestion	Mean Score	0	0.6	1.0	2.0^†^
	Prevalence	0/4	2/5	3/5	4/5
Hepatic vacuolation	Mean Score	3.0	3.0	2.6	1.8
	Prevalence	4/4	5/5	5/5	5/5

Tissues were scored semiquantitatively for histomorphological features. *Prevalence* indicates the fraction of examined animals displaying a histopathological feature. Bullets indicate histological observations in exposed animals that are statistically different from control at *p* ≤ .05 using the Kruskal-Wallis test. Daggers indicate specific conditions different from control at *p* ≤ .05 (uncorrected) in *post hoc* testing with the Wilcoxon ranks test. See Additional file 2 for images of stained sections and more detail

Unsurprisingly, skeletal muscle showed degenerative changes in exposed fish characterized by randomly distributed, segmental, coagulative sarcoplasmic disintegration. Although myodegenerative lesions are occasionally observed in otherwise healthy fish [32, 33], the preponderance and relative severity of such lesions in exposed fish were consistent with a concentration- dependent DDVP-induced effect (Table 1). There was degeneration in the testes of exposed fish, but it is unclear whether the testicular effects reflect a direct consequence of DDVP toxicity or a nonspecific stress response which has previously been reported in fish [34].

We observed that pancreatic zymogen granule stores were depleted in exposed fish. This effect has not previously been documented in zebrafish but has been seen in pancreata of other fish species under conditions of reduced caloric intake [35] and in viable human pancreas fragments exposed to the organophosphorus pesticide echothiophate [36].

Although the difference in the score for renal congestion between exposed and unexposed fish did not attain statistical significance, the prevalence of erythrocytic congestion in the sinsusoidal capillaries of the kidney increased with DDVP concentration (Table 1; Additional file 2). Similar findings have been reported in dogs exposed to DDVP where vascular congestion and hemorrhage presented in multiple organs [37, 38].

Exposed fish showed reduced hepatocellular vacuolation (Fig. 2a), suggesting that liver glycogen levels might be depressed as discussed below. Although the difference in the extent of vacuolation between exposed and control conditions was not statistically significant, there was a trend toward less vacuolation with increasing DDVP concentration (Table 1; Fig. 2a). In the low concentration condition, 80 % of the fish were scored as highly or moderately highly vacuolated, while in the high concentration condition 80 % were scored as minimally vacuolated (Table 1). To test whether the reduction in vacuolation might indicate a loss of glycogen, we stained fish sections for glycogen with Periodic Acid-Schiff (PAS). Glycogen staining was markedly diminished in the livers of fish exposed to the high concentration of DDVP (Fig. 2b) although there was little difference in PAS staining intensity between the low exposure group and controls. A diminution of liver glycogen stores has been described following exposure to other organophosphorus pesticides [39] and is concordant with the alterations in carbohydrate metabolism described below.

### Identification of biological processes affected by DDVP exposure

Among the 43,803 probes on the microarray, we found 22,585 (corresponding to 18,632 genes) that had a signal to noise ratio ≥ 3 in all replicates of at least one exposure condition, and selected them for further analysis. We identified differentially expressed transcripts in this set using a two-way analysis of variance (ANOVA; dose and exposure) with contrasts analysis for each concentration, a step-up Benjamini and Hochberg False Discovery Rate [40] of 0.001, and a fold change difference filter of ≥ 1.8 between exposed and control fish. To find biological processes affected by DDVP exposure, we performed unsupervised Gene Ontology (GO) enrichment analysis of the differentially expressed transcripts using the web-based tool GOTreeMachine [41], Ingenuity Pathway Analysis (IPA), and manually curated reference gene lists. These analyses suggested that multiple biological processes and networks are affected by DDVP exposure (Additional file 3). Major categories included *carbohydrate metabolism*, *lipid metabolism*, *detoxification*, and *proteasomal responses* (gene ontology terms are indicated by italic text).

### Reactive oxygen species stress and unfolded protein response pathways

**Fig. 3:**
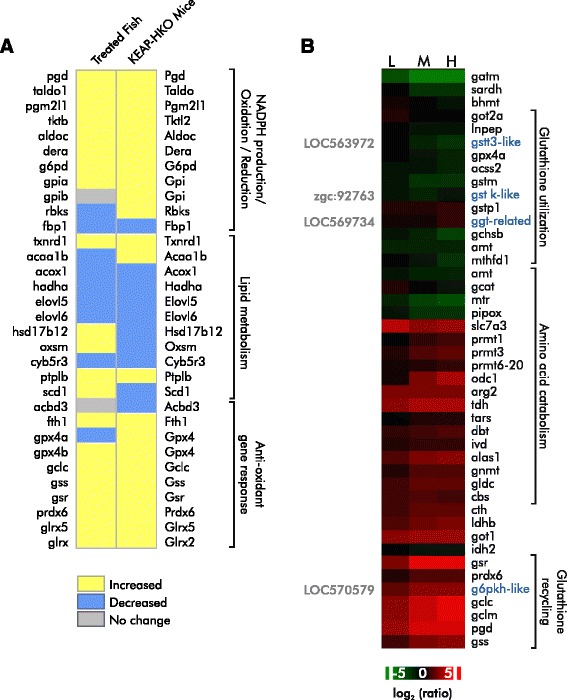
The expression of genes involved in Nrf2 signaling and glutathione metabolism is altered in zebrafish exposed to DDVP. **a** DDVP exposure and constitutive Nrf2 activation affect similar sets of genes. The heat map compares the direction of change in liver message levels in DDVP-exposed zebrafish and KEAP-HKO mice with constitutively active Nrf2 relative to unexposed fish and normal mice. Mouse and functional data are taken from [46]. Zebrafish gene symbols are to the left, and mouse symbols are to the right. We show genes that could be definitively mapped between zebrafish and mice and that met our criteria for expression (see Methods). RefSeq and probe IDs are presented in Additional file 6. **b** The heat map depicts changes in the expression of genes involved in glutathione metabolism and precursor biosynthesis. In a few cases, the association between a gene and a database could not be definitively made. In these instances, tentative characterizations are shown in blue on the right and the database identifier is shown on the left. Changes in gene expression are shown as the log_2_ ratio relative to control. L, M, and H indicate low, mid, and high exposure concentrations

Reactive oxygen species (ROS) have been implicated in DDVP toxicity by others [25, 42], potentially leading to Parkinsonism [42–44] and liver dysfunction [8, 25]. Our functional analysis revealed enrichment for processes involved in *glutathione metabolism*, suggesting that DDVP exposure affects ROS stress responses at the message level (Additional file 3). The transcription factor Nrf2 (nuclear factor [erythroid-derived 2]-like 2) is the “first responder” to reactive oxygen stress and a key regulator of transcriptional ROS stress responses [45]. Nrf2 is constitutively active in the liver of Keap 1-hepatocyte knockout mice (KEAP-HKO) [46], so to determine whether Nrf2 might be involved in the ROS response to DDVP, we compared the behavior of the set of genes differentially affected by the constitutive activity of Nrf2 in KEAP-HKO mice to matched genes in the fish. We observed considerable overlap in the gene expression profiles of the DDVP exposed fish and KEAP-HKO mice (Fig. 3a). The affected genes are associated with the ontology terms, *NADPH generation*, including the *pentose phosphate pathway* (PPP), *anti*-*oxidant response*, including *glutathione synthesis*, and *lipid biosynthesis*.

As part of its role in controlling cellular ROS responses, Nrf2 transcriptionally regulates the glutathione synthetase and glutamate-cysteine ligase network [47] to maintain cellular glutathione homeostasis, which is critical for cytoprotection in both physiological and pathological oxidative stress states. Consistent with activation of Nrf2 signaling, we observed increased expression of genes encoding crucial enzymes for biosynthesizing, processing, and recycling reduced and oxidized glutathione. Affected genes include glutathione reductase (*gsr*), glutathione synthetase (*gss*), and glutamate-cysteine ligase (*gclc*, *gclm*) as well as genes involved in metabolism of glutathione building blocks (glutamate, glycine and cysteine) (Fig. 3b). The levels of transcripts for enzymes essential for amino acid interconversions that contribute to glutathione biosynthesis were also upregulated, *got1* and *got2* (glutamine transamination), *cbs* and *cth* (serine to cysteine), *agxtl* (alanine to glycine), and *glulb*, *glul* and *gls2* (glutamine to glutamate). In contrast, transcripts for enzymes involved in the catabolism of GSH precursors were down-regulated: transcript levels for three of the four enzymes involved in the glycine cleavage system (*mthfd1*, *amt*, and *gcsh*) [48] were reduced. Although we did not measure the concentration of glutathione in DDVP exposed fish, the pattern of gene expression described above argues that glutathione synthesis increases in response to DDVP [49] to protect cells from elevated levels of ROS [50, 51].

**Fig. 4:**
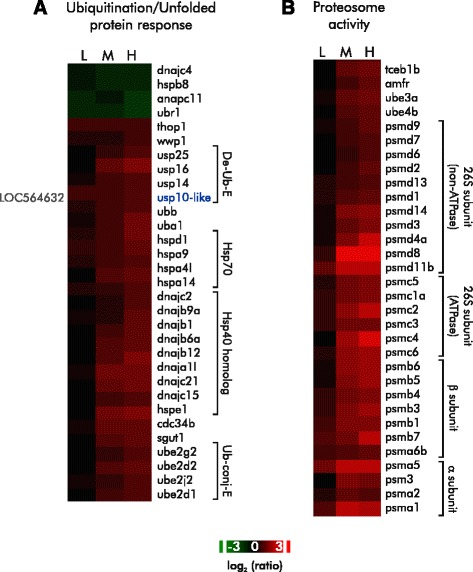
DDVP increases the expression of genes involved in ubiquitination, the unfolded protein response, and proteosome formation. Gene symbols are to the right. **a** The heatmap depicts the dose-dependent induction of genes involved in ubiquitination and the unfolded protein response. RefGene LOC564632 was tentatively identified as ubiquitin specific protease 10 **b**) The heatmap depicts the induction of genes for the components of the proteosome. Changes in gene expression are shown as the log_2_ ratio relative to control. L, M, and H indicate low, mid, and high exposure concentrations

The enriched GO-terms, *detoxification and metabolism*, s*tress response*, and *ubiquitination*/*proteasomal degradation systems*, include genes whose function is consistent with mitigating increased ROS (Fig. 4). For instance, transcripts of thioredoxin (*txnrd1*) and peroxiredoxin 1 (*prdx6*), which are known to reduce peroxides via the thioredoxin system, were increased as were messages for monooxygenase cytochrome P450 (*cyp2v*) and glutathione *S*-transferase (*gstp1*). Other Nrf2 responsive genes in the phase II detoxification and radical species processing pathways, including *cyp1a* (cytochrome P450 1A1) [52] and *ephx1* (microsomal epoxide hydrolase) [53], were also induced.

DDVP exposure also affects other responses downstream of Nrf2, such as the ubiquitin-proteasome system and unfolded protein response (UPR) (Fig. 4; for more discussion of proteosomal response see the section DDVP and Autophagy below), which often work in tandem to repair and remove proteins that have been damaged and misfolded consequent to increased ROS [54–57]. Here, induction of ubiquitin-UPR genes was clearly evident at the LC_20_ and LC_60_ levels (enrichment *p*-values: 3.51 × 10^−6^ and 6.91 × 10^−7^, respectively). The robust induction of these genes suggests that this system is active. Some of the well characterized genes induced include heat shock family members, the ubiquitin-conjugating enzymes, the adapter to E3 ubiquitin ligase, ubiquitin ligase E3 and the proteasome 26S, proteasome subunits of both non-ATPase and ATPase (PSM), UBC-a polyubiquitin precursor, UBA1-an enzyme essential to the first step of ubiquitin conjugation, and members of the ubiquitin E2 and E3 family.

Collectively, these observations support previous studies showing that DDVP induces ROS and activates Nrf2’s downstream effectors [10, 25, 42, 46]. The responses observed also argue for a potent anti-oxidant response involving the Nrf2 system including the thioredoxin and glutathione pathways, chaperones and stress responses, ubiquitination and proteasome degradation, and metabolic detoxification [49, 58, 59]

### DDVP and energy utilization

**Fig. 5:**
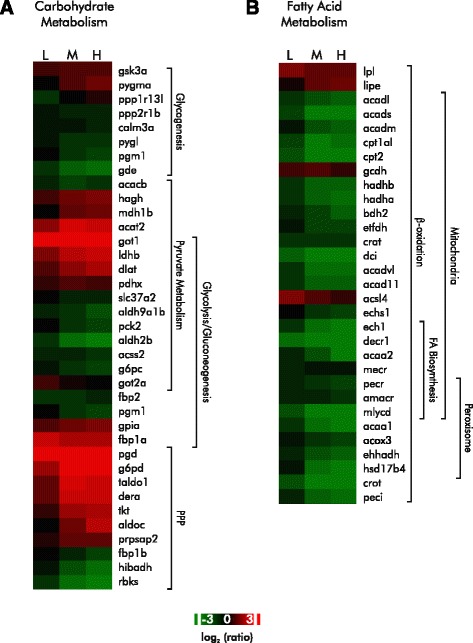
DDVP exposure alters expression of genes involved in carbohydrate and lipid metabolism. **a** Heatmap of the expression of genes involved in carbohydrate metabolism. **b** Heat map of the expression of genes involved in β-oxidation and fatty acid biosynthesis. Changes in gene expression are shown as the log_2_ ratio relative to control. Changes in gene expression are shown as the log_2_ ratio relative to control. L, M, and H indicate low, mid, and high exposure concentrations

**Fig. 6:**
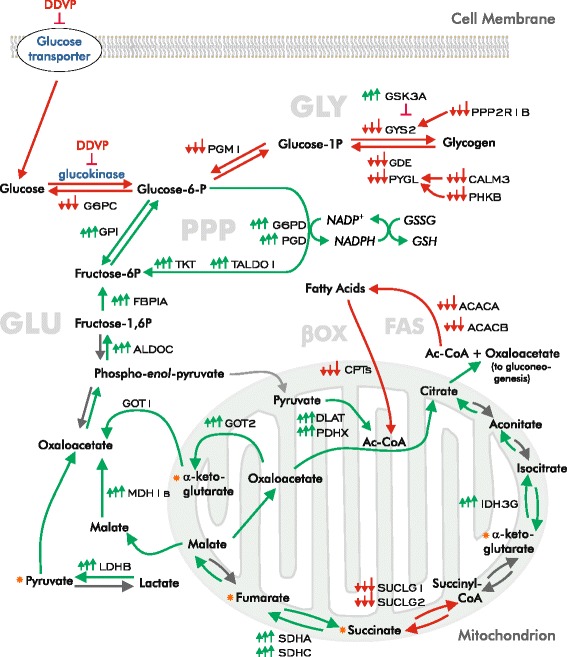
DDVP profoundly affects energy metabolism. The diagram illustrates the predicted effects of DDVP on carbohydrate metabolic processes. The effects of DDVP on glucokinase and the glucose transporter (shown in blue) are derived from the literature (see text). Clusters of small arrows adjacent to gene symbols (small caps) denote the direction of differential expression in exposed fish (red, down-regulation; green, up-regulation). Large arrows indicate the direction of biochemical processes or reactions. Green indicates a predicted increase, and red indicates a predicted decrease in the process. Gray arrows indicate no predicted change or an inability to predict a change. Proteins are shown in blue. Biological processes are labeled with large, pale gray capital letters. PPP: pentose phosphate pathway. βOX: βoxidation. FAS: fatty acid synthesis. GLU: gluconeogenesis. GLY: glycogenolysis/glycogen synthesis. Orange asterisks indicate points at which amino acids can enter carbohydrate metabolism by transamination. GSSG: oxidized glutathione. GSH: reduced glutathione. We excluded *fbp1b* from the metabolic map because its expression behavior appears to be confounded by high background transcription (data not shown)

Functional analysis of the differentially expressed genes in exposed fish revealed enrichment of GO terms for carbohydrate and lipid metabolism (Additional file 3). When we mapped the behavior of the differentially expressed genes associated with these terms (Fig. 5a and b) to biochemical pathways, we found evidence of large perturbations in carbohydrate and fatty acid metabolism. Figure 6 illustrates how the observed alterations in gene expression are proposed to affect carbohydrate metabolism. The transcript data suggest that there is a shift of sugar metabolism into the pentose phosphate pathway, and a substantial reduction in both *β* -oxidation and fatty acid synthesis.

#### The effects of DDVP on carbohydrate metabolism

DDVP exposure pervasively affected the expression of genes involved in glucose metabolism in the liver (Figs. 5a and 6). The alterations in gene expression we observed suggest that glycogen stores have been depleted and that extracellular sources of glucose are inadequate for hepatic needs. In response, the carbon flux is redirected into gluconeogenesis for energy and into the pentose phosphate pathway to generate reducing equivalents to mitigate oxidative stress. We observed a reduction in the levels of the transcripts for key enzymes involved in both glycogenolysis and glycogen synthesis. Message levels for glycogen phosphorylase (*pygl*), glycogen debranching enzyme (*gde1*), and phosphoglucomutase (*pgm1*) were all reduced as were the glycogenolytic regulatory enzymes calmodulin 3 phosphorylase kinase (*calm3a*) and phosphorylase kinase (*phkb*). The abundance of transcripts for protein phosphatase 2, regulatory subunit A-β (*ppp2r1b*) and glycogen synthase 2 (*gys2*), enzymes involved in glycogen synthesis, was reduced, while transcripts for the negative regulator, glycogen synthase kinase (*gsk3a*), increased in abundance. These observations are consistent with our histological data (Fig. 2) and are also consistent with previous reports that glycogen stores are reduced following exposure to DDVP and other organophosphorus pesticides [60, 61].

The abundance of transcripts for glycogenolytic enzymes was markedly reduced—presumably because of glycogen depletion. Yet glycogen phosphorylase enzymatic activity is acutely stimulated in DDVP-exposed rats [9, 62]. Since our analysis was performed 24 h after exposure began, the reduced transcript levels might be the result of a feedback response to the depletion of glycogen stores.

The presumptive reduction in glycogen synthesis may result from the direction of most, if not all, available glucose into the PPP at the expense of rebuilding glycogen stores. Glucokinase is known to be inhibited by DDVP [61] and there is some evidence that the activity of the glucose transporter is inhibited by DDVP [63], suggesting that gluconeogenesis is the main or a significant source of glucose.

Increased message levels for key enzymes involved in both *de novo* glucose synthesis and the PPP suggest that newly synthesized glucose is being directed to the PPP to fulfill the basic energy needs of the cell and provide reducing power for free radical removal and glutathione-mediated detoxification [46, 64, 65]. Consistent with an increased requirement for reducing equivalents in response to DDVP intoxication, increased transcription of messages for key PPP enzymes, including glucose-6-phosphate dehydrogenase (*g6pd*), transketolase (*tkt*), transaldolase (*taldo1*), and phosphogluconate 2-dehydrogenase (*pgd*) were observed (Figs. 5a and 6). Increased transcription for fructose-1-6-*bis*phosphatase (*fbp1a*), the rate-limiting enzyme in gluconeogenesis, is consistent with the prediction that glucose is being supplied to the PPP by *de novo* synthesis.

DDVP also affects the abundance of transcripts for key players in the malate/aspartate shuttle (the glutamic-oxaloacetic transaminases *got1* and *got2a*, and malate dehydrogenase 1b, *mdh1b*) which provides gluconeogenic precursors as well as transferring reducing equivalents between the cytoplasm and mitochondrion for electron transport, oxidative stress defense, and efficient utilization of lactate in the liver [66, 67]. Increases in the transcript levels of these enzymes may provide a counterbalance to both an accumulation of circulating lactic acid following DDVP exposure [9, 68] via the Cori cycle. The observed increased transcript levels of the dihydrolipoyl transacetylase and pyruvate dehydrogenase (*dlat* and *pdhxp*) subunits of pyruvate dehydrogenase and lactate dehydrogenase B4 (*ldhbl*) are consistent with this conjecture.

The pattern of transcriptional expression of key tricarboxylic acid (TCA) cycle enzymes also suggests a shift toward gluconeogenesis as indicated in other studies [69]. The reduction of transcript abundances for succinate-CoA ligase, GDP-forming α and β subunits (*suclg1* and *suclg2*) and the increase of isocitrate dehydrogenase 3γ (NAD+) (*idh3g*) and succinate dehydrogenase complex (*sdha* and *sdhc*)] suggest that the TCA cycle is shifted toward the accumulation of citrate and oxaloacetate. Since citrate induces inhibitory feedback against phosphofructokinase (PFK1), the rate-limiting enzyme in glycolysis, gluconeogenesis likely predominates under these conditions. Consistent with this scenario, others have demonstrated that PFK1 activity is repressed by DDVP [9]. Increased fructose bis-phosphatase 1 (*fbp1a*) mRNA levels were observed upon DDVP exposure. Together, these results suggest that DDVP exposure modulates carbohydrate metabolism to generate reducing equivalents from *de novo* synthesized glucose through the PPP in response to oxidative stress. These changes in message abundance are also consistent with previously observed DDVP exposure effects on glucose metabolism in other models [9, 63, 70].

#### The effects of DDVP on lipid metabolism

DDVP exposure induced a general perturbation of fatty acid metabolism as reflected in the down-regulation of transcripts associated with the GO terms, *β*-*oxidation*, *fatty acid synthesis*, *fatty acid modification*, *moiety attachment to transport*, and *PPAR signaling* (Additional file 3, Functional analysis of differentially expressed genes). Down-regulation of fatty acid synthesis is consistent with our presumption that the availability of exogenous and stored carbohydrates is limited. Indeed, transcript levels for enzymes involved in fatty acid synthesis and elongation were reduced, suggesting that this process was restricted (Fig. 5b). We expected to find evidence of a shift in energy metabolism toward β-oxidation, and in fact, up-regulation of genes involved in processing and transporting fatty acids and derivatives (acyl-CoA synthetase long-chain family member 4 [*acsl4*], hormone-sensitive lipase [*lipe*], and lipoprotein lipase [*lpl*]) (Fig. 5b) was observed. But contrary to expectation, the abundance of transcripts for virtually every step of the β-oxidation of fatty acids was down-regulated in the mitochondrial and the peroxisomal compartments.

A complete rationale for this marked reduction in message levels is not entirely evident and could be a consequence of any or all of the following mechanisms: a response to elevated levels of ROS; the result of mitochondrial damage; an increase in transcripts of negative regulators of β-oxidation; or inhibitory feedback by increased intracellular citrate. In support of a role for Nrf2, genes involved in both fatty-acid synthesis and β-oxidation are repressed in the KEAP-HKO mouse [46] (Fig. 3a). As expected from the overlap of differentially expressed genes in the knockout animal and the DDVP-exposed fish, a similar group of pathways is also enriched in the mouse knock-out experiment and in our study.

### Autophagy and apoptosis

#### DDVP exposure induces the expression of autophagy essential genes

**Fig. 7:**
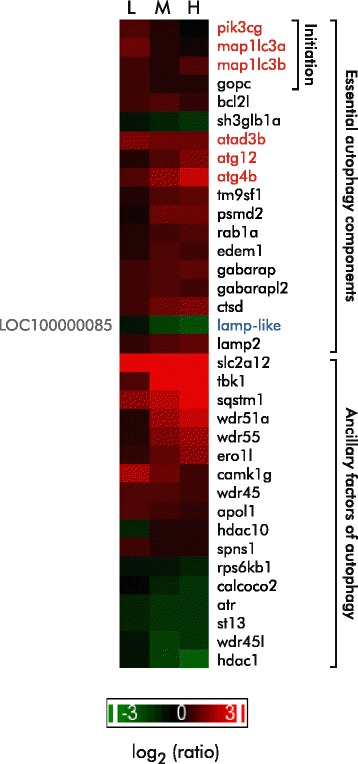
DDVP-dependent alterations in the abundance of transcripts involved in autophagy. The heatmap depicts the dose-dependent alterations in the abundance of genes involved in autophagy. The gene symbols for core autophagy machinery genes are shown in red. RefGene LOC100000085 was tentatively identified as a *lamp*-like gene. Changes in gene expression are shown as the log_2_ ratio relative to control. L, M, and H indicate low, mid, and high exposure concentrations

Functional enrichment analysis of differentially expressed genes suggests that DDVP exposure induces autophagy (enrichment *p*-values: 2.52 × 10^−7^ [low], 9.54 × 10^−3^ [mid], 3.00 × 10^−2^ [high]), and the expression levels of genes critical for autophagy increase modestly with increasing concentrations of DDVP (Fig. 7), including the core autophagy machinery genes and autophagy ancillary factors (Fig. 7). Even at the lowest concentration of DDVP, there was an increase in abundance of transcripts for the core autophagy enzymes including p110γ (*pi3kcg*), microtubule-associated proteins 1A/1B light chain (*map1lc3a* and *map1lc3b*), autophagy related cysteine proteases (*atg4b*, *atg12*) and the Golgi-associated PDZ scaffold protein C (*gopc*). Moreover, the levels of mRNA for enzymes crucial to the maturation of autophagolysosomes such as *wdr45*, *wdr45l*, *lamp*-*2*, *gabarap*, *gabarapl2*, *tm9sf1*, and *rab1a* were increased.

In addition to transcripts encoding molecules classically involved in autophagy, we observed altered message levels for a number of ancillary factors of autophagy identified in a yeast two hybrid screen [71] (Fig. 7). These proteins are involved in a number of biological processes associated with autophagy including ubiquitination, lipid/fatty acid transport, and apoptosis.

The driving force behind the induction of autophagy in this experiment is uncertain. Autophagy is characterized by the sequestration of long-lived proteins, oxidized lipids, and supernumerary or damaged organelles to vacuoles with subsequent catabolism [72, 73]. However, in recent years, it has become clear that autophagy not only provides cytoprotection from damaged cellular components but also access to an internal reservoir of molecules for biosynthesis and energy generation during nutrient restriction [74]. We noted earlier that there was strong induction of the UPR, and it now clear that proteosomal activity is also integrated with autophagy and provides a significant contribution to proteostasis under some conditions [75, 76].

The alterations in the expression of genes involved in energy metabolism that we discussed above suggest that exposed fish may be deriving their energy from catabolism of preformed cellular constituents since other sources of energy are not available. Potential entry points for amino acids into gluconeogenesis are indicated by asterisks in Fig. 6. Alternatively, or in addition, the autophagic and proteosomal responses may be directed at clearing cellular components damaged by ROS and preventing toxicity arising from the accumulation of damaged organelles [75–78].

#### DDVP exposure and the expression of apoptosis-related genes

Gene ontology enrichment analysis indicated that genes associated with the term *apoptosis* occurred more frequently than expected by chance (enrichment *p*-values: 7.48 × 10^−1^ [low], 7.55 × 10^−8^ [mid], 3.31 × 10^−4^ [high]), and DDVP has been shown to induce apoptosis in neuronal cells because of impaired mitochondrial bioenergetics [42]. However, we found no histological evidence of apoptosis in the liver. The expression pattern of the enriched genes was complex. For example, both concentration dependent down-regulation of mRNA levels of anti-apoptotic components (*bcl2*, *bcl2l*, and *mcl1a*) and up-regulation of the pro-apoptosis components of the intrinsic pathway (*bax*, *apaf1*, and *casp9*) were observed. On the other hand, transcripts of the tumor necrosis factor receptor superfamily member 14 (*tnfrsf14*) and TNF-α (*tnfa*) mRNA of the extrinsic pathway were both down-regulated. Interestingly, the *tnfrsf6b* mRNA level was also induced by DDVP exposure. In tumor cells its expression is negatively correlated with apoptosis [79]. Based on the evidence in hand, we propose that cellular stress related to ROS generation may have primed apoptotic pathways, but that the cells of the liver have not yet committed to cell death.

### Role of nutrient deprivation in the response to DDVP

We observed aberrant locomotion and accumulation of food in the tanks of animals exposed to the mid and high concentrations of DDVP, raising the possibility that the exposed fish fed poorly and experienced nutrient restriction. We also observed depletion of zymogen granules in the pancreata of exposed fish which might have impaired digestion (Table 1). When Drew and colleagues [80] deprived female zebrafish of food for 21 days and examined gene expression in the livers of the fish in a microarray experiment, they observed that the starved fish showed patterns of gene expression related to ROS responses and energy metabolism similar to those that we observed in DDVP exposed fish. They also observed down-regulation of transcripts for proteins involved in both fatty acid synthesis and β-oxidation.

To clarify whether nutrient deprivation was important for the effects of DDVP exposure in our experiments, we tested the effect of limiting feeding on gene expression. In our usual experimental design, on the day that the exposure begins, fish are fed once before the exposure begins and then again several hours later. Control fish are fed at the same times. Twenty-four hours after the beginning of the exposure the fish are harvested. If DDVP affects feeding by the fish and/or adversely affects nutrient processing, then the fish may not derive the full nutritional benefit of feeding during the experiment. Therefore, we tested the effect of three feeding protocols on gene expression. We used 20 fish in each condition. In the control condition, fish were fed throughout the experiment, and the final feeding occurred 4 h before harvest. In a condition designed to evaluate the effect of the fish entirely missing feeding because of the effects of DDVP, normally scheduled feedings were omitted, resulting in an approximately 29 h period before harvest in which the fish received no food. In the third condition, the fish were not fed for approximately 50 h in an effort to account for possible synergism between the effects of DDVP exposure and nutrient deprivation. We prepared microarrays from the experimental fish and analyzed them together with the original DDVP exposure data. Using the set of 18,658 expressed genes from the DDVP analysis, we identified 5481 differentially expressed genes with an FDR ≤ .001 in a two-way ANOVA (batch, and exposure, including the DDVP and nutrient deprivation conditions). We did not filter based on fold change in this comparison.

**Fig. 8:**
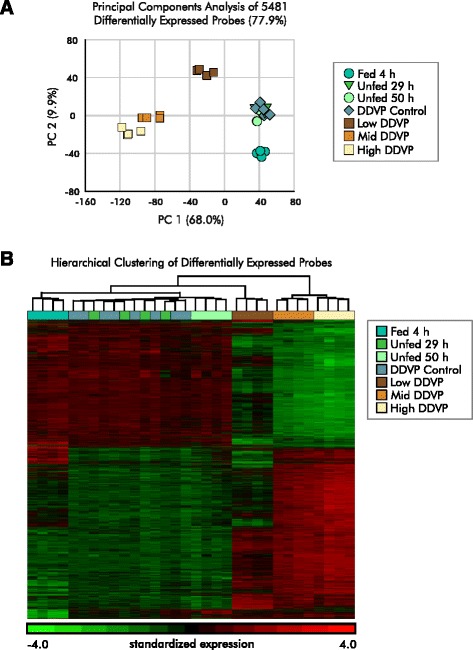
Effects of DDVP on liver gene expression are distinct from the effects of nutrient restriction. The batch effect due to different experimental dates for DDVP exposure and nutrient deprivation was removed using the batch effect removal tool in Partek Genomics Suite before plotting and clustering. **a** Principal components analysis (PCA) of 5481 genes differentially expressed in the livers of nutrient restricted or DDVP-exposed fish in comparison with DDVP exposure experiment controls (see text). Nutrient deprived fish form a tight cluster with controls from the DDVP exposure experiment that is well separated from DDVP-exposed fish. The percentage of variance explained by the PCA is shown in parenthesis. **b** Heat map of the expression levels of the 5481 differentially expressed genes in DDVP-exposed and nutrient deprived fish. Rows are individual genes and columns are pools of 4–5 animals. Expression levels for each gene were standardized to a mean of zero and a standard deviation of one

No large differences in gene expression between the unexposed control fish from the DDVP arm of the experiment and the nutrient deprived fish were obvious (Fig. 8a and b). Although the fed fish form a distinct cluster in the principal components plots, they are clearly more similar to the DDVP exposure controls and nutrient deprived fish than to the DDVP exposed ones. To confirm this conclusion, we directly compared the expression of a subset of the genes across the nutrient deprivation and DDVP experiments using the QuantiGene® multiplex gene expression assay platform (Additional file 4). We selected genes involved in the ROS response, autophagy, and carbohydrate and fatty acid metabolism. Only minimal differences were observed between the control animals and the nutrient deprived ones, and DDVP exposure produced distinct responses. We conclude that nutrient deprivation does not play a large role in the alterations in gene expression we observed in DDVP exposed fish.

### Causes of oxidative stress

Although there is abundant evidence that DDVP exposure results in oxidative stress [3], the underlying mechanism has not been well elucidated. DDVP is metabolized by both glutathione-dependent and glutathione-independent pathways [15–17]. Consistent with our observations at the message level, the activities of a number of enzymes involved in glutathione synthesis and recycling (glutathione reductase, glutathione-*S*-transferase, glutamate:cysteine ligase) are known to be increased concomitant with an increase in glutathione content in the liver of DDVP exposed fish [49]. Since the animals in our study are constantly exposed to DDVP by immersion, it is plausible that DDVP detoxification depletes and/or strains the capacity of the glutathione biosynthetic and regenerating systems, resulting in elevated ROS simply from normal metabolic activity.

DDVP exposure also reduced the transcript abundance of key enzymes involved in glyoxal detoxification, alanine-glyoxylate aminotransferase (*agxtl*), aldo-keto reductase family 1, member A1 (*akr1a1a*) [81], and glyoxalase (*glo1*) (not shown). Glyoxal has been suggested to be a toxic metabolite of DDVP [15–17, 82] and has the potential on its own to induce cell damage, deplete GSH, generate ROS, collapse the mitochondrial membrane potential, induce lipid peroxidation, and produce formaldehyde[4, 15–17, 25, 42, 83]. Although direct evidence of glyoxal and its metabolites in tissues exposed to DDVP remains elusive [16], it is conceivable that glyoxal or its metabolites could exacerbate DDVP toxicity.

There is also some evidence that DDVP perturbs Ca^2+^ homeostasis which is important in controlling responses to oxidative stress [84, 85]. DDVP exposure altered the transcript abundance of the calcium storage and signaling pathway proteins, Pp3cc (protein phosphatase 3, catalytic subunit γ isozyme), calreticulin-like 2, and aspartate β-hydroxylase, calmodulin-3, calcium/calmodulin-dependent protein kinase IG, Atf4 (activating transcription factor 4), and Creb1(cyclic amp responsive element binding protein 1) (not shown). These changes are consistent with results showing that DDVP exposure affected calcium homeostasis in the plasma of carp [86], increased free Ca^2+^ in rat brain stem, cerebrum, and cerebellum [87], and increased mitochondrial Ca^2+^ uptake in hepatic mitochondria [87].

DDVP exposure appeared to induce damage in the kidney (Table 1), the central organ in the regulation of calcium homeostasis. Histopathological changes in the glomeruli, proximal convoluted tubules, distal convoluted tubules, and collecting tubules were observed in DDVP treated mice [88]. Kidney injury could compromise calcium homeostasis and result in misregulation of responses to elevated cellular RO [88, 89].

## Conclusion

To our knowledge this is the first comprehensive evaluation of the effects of DDVP on gene expression in the liver. While a number of prior studies described the effects of DDVP exposure on hepatic metabolism [25, 26], they focused on particular metabolites and/or the activities of particular enzymes. Our analysis draws together many of the results of these prior studies and extends them. We observed coordinated changes in the expression of a large number of genes involved in energy metabolism and cellular responses to increased ROS levels. These results argue that an appreciable part of the effects of DDVP on energy metabolism and oxidative stress is transcriptionally regulated and suggest that the transcriptional regulator Nrf2 is a key player in these events.

It is well established that exposure to DDVP and other organophosphorus pesticides results in reactive oxygen stress, injury to mitochondria, lipid peroxidation and other events consistent with elevated ROS levels [26]. However, the cause of the elevated oxidative stress is not known.

DDVP can be detoxified via a glutathione-dependent pathway and can also produce glyoxal which can deplete glutathione [15–17]. DDVP exposure has been associated with diminished glutathione levels in the mitochondria [25]. When the route of exposure is immersion, the cumulative effect may be to drastically increase ROS because of glutathione depletion. We also observed alterations in transcripts for proteins involved with Ca^2+^ regulatory pathways which are also involved in the regulation of responses to ROS and could contribute to overall DDVP toxicity.

To highlight fundamental biochemical processes rather than inter-individual variability, we deliberately limited the scope of the study by studying pools of male fish. The use of male fish reduced potential inter-probe interference and cross-hybridization from the highly abundant egg protein messages in mature female fish [90], and the use of pools damped out individual variation. Key aspects of the model we propose, however, can now be easily tested across individual fish and in female animals using either the set of Quantigene probes we developed or other analytical approaches appropriate to particular studies based on our observations.

While it is true that transcript analysis alone cannot provide or confirm a comprehensive explanation of cellular events, in aggregate, the data we report suggest that DDVP exposure elicits a highly coordinated response tightly tied to defenses against intracellular ROS insult in the liver.

## Methods

### Animal studies

#### Husbandry

Mature adult (6–12 months of age) male zebrafish, *Danio rerio* (Tübingen strain) were used in this study. Because egg protein transcripts are extremely abundant in the livers of breeding females and might confound the microarray analysis, only male fish were used. Any female fish inadvertently collected were removed after being identified by visual inspection. To identify occasional females that could not be visually distinguished from males, we tested all RNAs by qPCR for the presence of vitellogenin transcripts which are not normally expressed in male fish [90, 91]. Zebrafish were maintained and tested at 25 °C with a 14:10 h (light/dark) photoperiod in 5-gallon glass aquaria adapted for flow-through use (60 mL/min; 5.4 turnovers/day). Fish were fed twice per day (1X flake food, 1X brine shrimp).

Research was conducted in compliance with the Animal Welfare Act, and other Federal statutes and regulations relating to animals and experiments involving animals and adheres to principles stated in the Guide for the Care and Use of Laboratory Animals [18] in facilities that are fully accredited by the Association for Assessment and Accreditation of Laboratory Animal Care, International. All procedures were reviewed and approved by the US Army Center for Environmental Health Institutional Animal Care and Use Committee.

#### Exposure study

DDVP was obtained from Chem Services, Inc. (CAS number: 62–73–7; West Chester, PA), and stock solutions were directly introduced into the aquaria containing zebrafish using a flow-through diluter system that maintained essentially constant concentrations of toxicant in the aquaria. Range finding experiments were also conducted using the flow through system at 4, 6, 10, 15, 16, 22, 25, 31, and 45 mg/L DDVP for a 96 h duration to estimate the 96 h LC_20_ (concentration for 20 % mortality) and LC_60_ (concentration for 60 % mortality; Additional file 1). To evaluate the response to the toxicant without producing mortality, fish were exposed to DDVP using a no-observed-effect concentration, the 96 h LC_20_, and 96 h LC_60_ for 24 h (target concentrations 6, 19, and 32 mg/L). The measured concentrations in the exposure experiment were 6, 16, and 30 mg/L, referred to as the low, mid, and high concentrations respectively. Fish were euthanized by immersion in MS-222 solution (0.5 g/L, pH 7.2; Sigma-Aldrich, St. Louis, MO). DDVP concentrations varied by approximately 1 % between the pre- and post-exposure samples. The aquaria were routinely inspected for dead and/or moribund fish. Dead fish were removed from the aquaria. Exposed fish were observed for altered behavior, distress signals, or signs of toxicity. Whole fish (5/condition) were preserved in Davidson’s solution for histological studies. Fish livers, brains, and hearts (20/condition) were collected and flash frozen in liquid nitrogen for future analysis. DDVP concentrations in the test tanks were verified prior to the initiation of the exposure and at the time of the termination of the study using a minor variation of EPA method 8141A and a Hewlett-Packard model 6890 gas chromatograph equipped with an electron capture detector and a Hewlett-Packard model 7673 auto sampler (Santa Clara, CA).

#### Nutrient restriction study

For the nutrient restriction study, fish were handled exactly as they were for the DDVP exposure study except that the nutrient restricted fish were fasted either for 29 or for 50 h before harvest (see text). The control fed fish received flake food 4 h before harvest.

#### Histology and tissue scoring

Fixed whole fish were embedded in blocks, sectioned transversely and stained with hemotoxylin and eosin. Histomorphological features were scored semiquantitatively by a pathologist (Experimental Pathology Laboratories, Inc., Sterling, VA) as 0, no observed effect; 1, a minimal effect; 2, a slight effect; 3, a moderately severe effect; and 4, a severe effect. Although five fish from each condition were harvested for histopathological assessment, one control fish proved to be a female and was excluded from analysis.

#### Glycogen quantitative analysis and tissue scoring

Periodic Acid-Schiff staining and evaluation of liver slices was also performed by Experimental Pathology Laboratories. To determine the relative amount of glycogen in the cytoplasm of hepatocytes, a pair of consecutive sections of whole fish were stained for either the periodic acid–Schiff (PAS) reaction or PAS plus diastase (an enzyme that breaks down glycogen). The PAS plus diastase slide serves as a negative control for the glycogen determination. Using bright field microscopy, the slides were examined by the pathologist and graded according to the relative amount of PAS-positive glycogen staining (evident as deep magenta granular stippling) that was present in the cytoplasm of hepatocytes, particularly, in the vacuoles. The sections were graded from 0 to 4 where 0 is none or minimal staining and 4 indicates the highest staining intensity. The Kruskal-Wallis statistic was used to determine statistical significance with a Wilcoxon *post hoc* test.

#### Cholinesterase assay

Four brains and hearts for each exposure condition were homogenized, and the supernatants were assayed for cholinesterase activity using the Ellman method [92] with 5,5’-dithio-bis(2-nitrobenzoic acid) (DTNB) and acetylthiocholine iodide (ASChI) as the substrate. Samples containing only 5,5’-dithio-bis-2-nitrobenzoic acid (Sigma, St. Louis, MO) were used as blanks. Hydrolysis of acetylthiocholine iodide (Sigma) was determined by the change in absorbance at 405 nm. To inhibit pseudocholinesterase activity, 100 μM tetraisopropyl pyrophosphoramide (iso-OMPA) was included in the assay. Data were normalized using protein concentration as determined using the BCA assay according to the manufacturer’s directions (Pierce, Rockford, IL). The Kruskal-Wallis test was used to identify significant exposure effects; if significant effects were identified (*p* < 0.05), *post hoc* analyses were performed using Tukey’s HSD test.

### Microarray analysis

#### RNA processing

Total RNA was extracted from livers by homogenization in Trizol® (Invitrogen, Carlsbad, CA) followed by a further purification using RNeasy® Mini kits (Qiagen, GmbH, Germany) according to the manufacturer’s protocols. The quality and quantity of total RNA were evaluated using an Agilent Bioanalyzer 2100 (Agilent, Santa Clara, CA) and the NanoDrop ND-1000 Spectrophotometer (NanoDrop, Wilmington, DE).

#### Microarray hybridization

Equal masses of total RNA from four or five fish were pooled to form one replicate. Four replicates per exposure condition (totaling 4 microarrays and 20 fish per replicate) were processed for hybridization to Agilent Zebrafish Oligo Microarrays (V2; part 019161) using the Low RNA Input Linear Amplification kit, Gene Expression Hybridization kit, RNA Spike-In kit, Stabilization and Drying solutions and the Gene Expression Wash Buffer kit (Agilent Technologies, Inc. Santa Clara, CA) according to the manufacturer’s protocol. Hybridization was performed according the recommendations by the microarray manufacturer. Scanning of the DDVP exposure slides, was performed using an Axon GenePix 4200 AL scanner with GenePix® software (Molecular Devices, Union City, CA) with a scan resolution of 5 μm, standard green filter, PMT gain set at 400 and scan power set to 30 % (first) then 100 % (second). These GenePix scans were combined such that any probes which were offscale in the 100 % scan (>32,000 units) were replaced by probes from the 30 % scan. Slides for the nutrient restriction study were scanned immediately after washing using an Agilent C2505C scanner following protocol GE1-107-Sep09 with default settings.

#### Statistical analysis

Microarray data were processed using Partek Genomic Suite software (Version 6.4 Copyright 2009, St. Louis, MO). Unprocessed median signal intensity data from the microarrays were quantile normalized and then log_2_ transformed. Outliers in the microarray data were identified by a principal components analysis (PCA) in Partek 6. To verify reproducibility, a pairwise correlation analysis was performed and inter-replicate dot plots of all probes were examined. Replicate arrays were only accepted if they had an *R*^*2*^ ≥ 0.95 without gross deviations from linearity on the dot plot. If a sample did not meet these criteria, a new microarray was processed from the total RNA. Only probes with a signal to noise ratio ≥ 3 in all four replicates of at least one condition were selected for further analysis. This filter produced a subset of 22,593 probes from the original 43,803 on the microarray. This subset was analyzed by 2-way Analysis of Variance (ANOVA, dose and exposure) with contrast analysis for each concentration to identify individual probes that were differentially expressed between each exposure group and its respective control. Probes with a step-up Benjamini and Hochberg False Discovery Rate [40] ≥ 0.001 for the concentration variable and a fold change greater than or equal to 1.8 from control in at least one treatment condition were retained for analysis.

#### Gene ontology analysis

Unsupervised Gene Ontology (GO) enrichment analysis was performed using the web-based tool GOTreeMachine (GOTM; http://bioinfo.vanderbilt.edu/webgestalt/) [41] and further validated by Ingenuity Pathway Analysis (IPA, Redwood City, CA) and Metacore (GeneGo, St. Joseph, MI) to identify biological processes being affected by the genes in this data set. For GOTM analysis and Metacore pathway analysis, background reference sets comprised the set of all probes with SNR ≥ 3. GO terms that are statistically over-represented in each exposure compared to the reference set were determined using a hypergeometric test with *p*-values adjusted using the Benjamini and Hochberg FDR (α = 0.05) with a threshold for the minimum number of genes per category set at four (*n* = 4) [93].

Since IPA analyses only support mammalian genes, only zebrafish genes with identified human homologues in IPA were used for this analysis. Manually curated reference gene lists for autophagy and apoptosis were also used. Briefly, comprehensive gene lists for autophagy and apoptosis were compiled from the literature and the on-line databases WebGestalt (http://bioinfo.vanderbilt.edu/webgestalt/), KEGG pathways (http://www.genome.jp/kegg), autophagy database (http://www.tanpaku.org/autophagy/overview.html), Human Autophagy Database-Public Research Centre for Health (http://autophagy.lu), The Apoptosis Database (http://deathbase.org), IPA, and Metacore. The gene lists were subsequently annotated with zebrafish homologues using Ensembl Biomart (http://useast.ensembl.org/index.html) or Zfin (http://zfin.org).

The curated gene set for autophagy and apoptosis was used to tabulate the count of associated transcripts for each exposure condition (high, mid, low) with the 22,593 probe-set used as a background for subsequent calculation. A hypergeometric test was used to test the significance of enrichment. We also examined individual gene responses within the enriched pathways and processes to determine whether the changes in their expression were consistent with predicted pathway activities.

### QuantiGene® analysis

A custom QuantiGene® gene expression array (Affymetrix, Fremont, CA) was designed by Affymetrix using transcript sequence identifiers matching probes of interest provided by the authors. This array was used to independently verify the expression patterns of representative genes in nutrient-deprived and DDVP exposed fish. The target sequences included two housekeeping genes (β-actin, and ribosomal protein L4) and 13 representative genes for the ROS response, carbohydrate and lipid metabolism, and autophagy (Additional file 5). The probes are now available from Affymetrix. Pooled zebrafish total RNA samples (see above) were processed following the Affymetrix QuantiGene Plex 2.0 Assay Manual using a Bio-Rad Bio-Plex 200 instrument (Bio-Rad, Hercules, CA). The plate was incubated overnight at 4 °C following the standard processing procedure and rewashed with SAPE Wash Buffer (Affymetrix) per the manufacturer’s instructions before scanning. The settings on the Bio-Plex instrument were Sample Size 100 μl; Timeout 45 s; DD Gate 5000–25,000; Bead Events/Bead Region 100.

Fluorescence readings from blank wells were subtracted from the fluorescence values for each transcript of interest. These resulting values were then normalized against the geometric mean of the expression of the two housekeeping genes for each sample. The geometric mean of the fold difference from the DDVP control was calculated and is presented in Additional file 4 .

### Availability of data and materials

Microarray data have been submitted to GEO (http://www.ncbi.nlm.nih.gov/geo/) with the accession number GSE66257.

## Additional files

Additional file 1: Figure S1. The mortality curve for zebrafish exposed to DDVP for 96 h was determined in range finding studies. (PDF 813 kb) Additional file 2: Figure S2. Effects of DDVP exposure on non-neural tissues. (PDF 7021 kb) Additional file 3: Table S1. Functional analysis of differentially expressed genes. (PDF 2394 kb) Additional file 4: Table S3. Replication of microarray data using Quantigene assays. (PDF 1546 kb) Additional file 5: Table S4. Gene, sequence and probe information for Quantigene probes. (PDF 1561 kb) Additional file 6: Table S2. RefSeq IDs for Fig. 3a. (PDF 26 kb)

## Abbreviations

AChE: Acetyl cholinesterase
ANOVA: Analysis of variance
bp: Base pairs
ChE: Cholinesterase
DDVP: Dichlorvos
GO: Gene ontology
GOTM: GOTreeMachine
GSH: Reduced glutathione
GSSG: Oxidized glutathione
IPA: Ingenuity Pathway Analysis
KEAP-HKO: Mice that have been genetically modified to show constitutive activation of Nrf2
KEGG: Kyoto Encyclopedia of Genes and Genomes
PAS: Periodic acid/schiff stain
PCA: Principal components analysis
ROS: Reactive oxygen species
SEM: Standard error of the mean
UPR: Unfolded protein response
