# Supplementary material for: Dichlorvos exposure results in large scale disruption of energy metabolism in the liver of the zebrafish, Danio rerio
Source: BMC Genomics. 2015 Oct 24;16:853. doi: 10.1186/s12864-015-1941-2 (PMC4619386; doi:10.1186/s12864-015-1941-2)
Supplement: Additional file 1: Figure S1. — The mortality curve for zebrafish exposed to DDVP for 96 h was determined in range finding studies. (PDF 813 kb) [file 12864_2015_1941_MOESM1_ESM.pdf]

Additional file 1

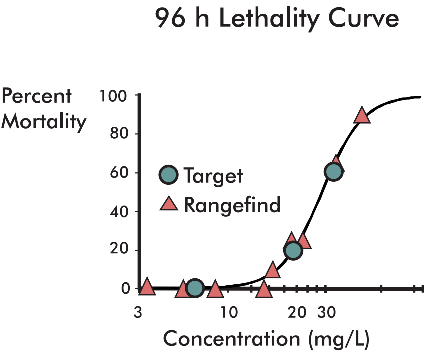

Figure S1. The mortality curve for zebrafish exposed to DDVP for 96 hours was determined in rangefinding studies. Circles indicate the target concentrations selected for the definitive experiment (Low, 6 mg/L; Mid/LC_{20}, 19 mg/L; High/LC_{60}, 32 mg/L).
