# Supplementary material for: Dichlorvos exposure results in large scale disruption of energy metabolism in the liver of the zebrafish, Danio rerio
Source: BMC Genomics. 2015 Oct 24;16:853. doi: 10.1186/s12864-015-1941-2 (PMC4619386; doi:10.1186/s12864-015-1941-2)
Supplement: Additional file 2: Figure S2. — Effects of DDVP exposure on non-neural tissues. (PDF 7021 kb) [file 12864_2015_1941_MOESM2_ESM.pdf]

Additional file 2

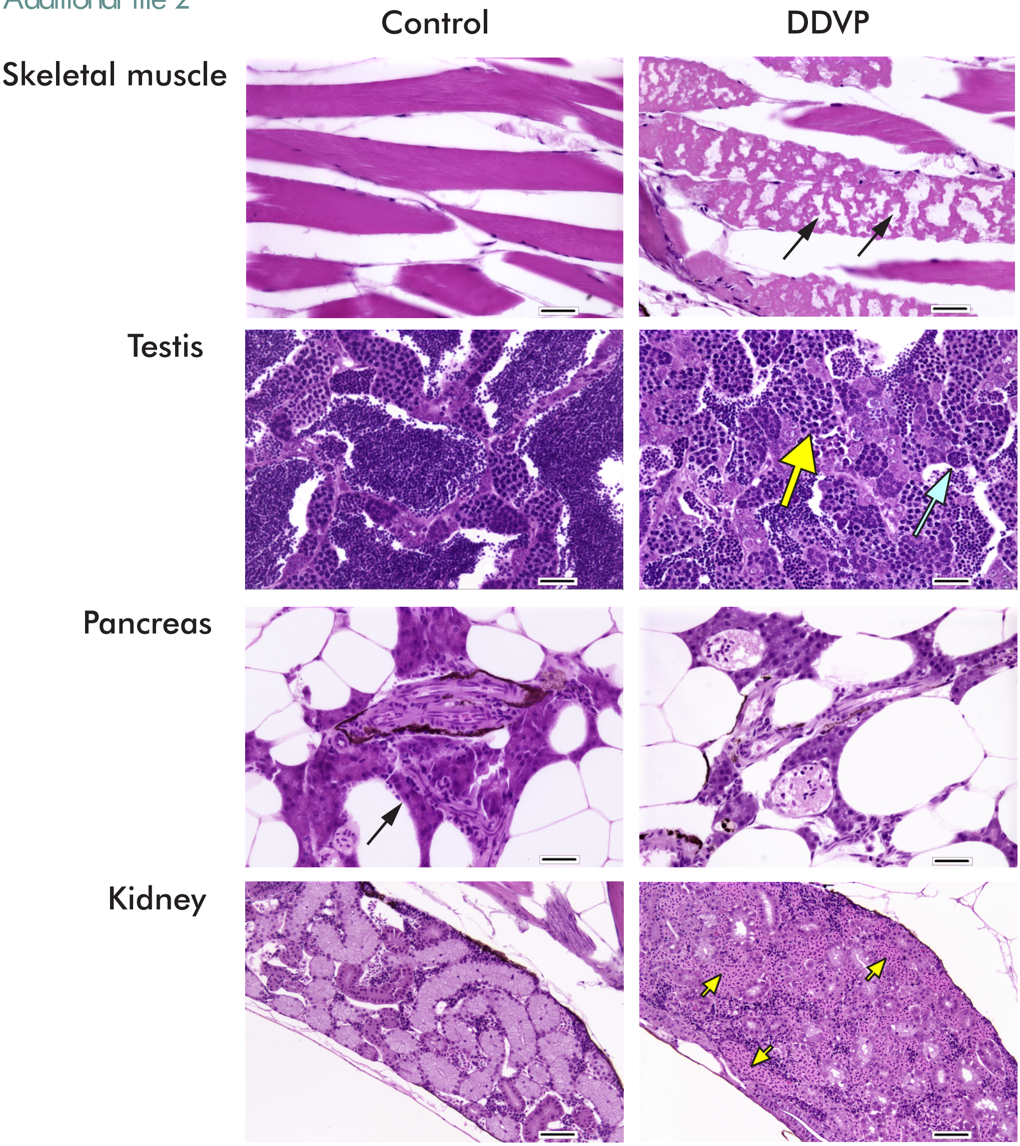

Figure S2 Effects of DDVP exposure on non-neural tissues. Representative images of hematoxylin and eosin stained transverse tissue sections are shown. Bars are 25 μm except for kidney which are 50 μm. ***Skeletal muscle***: Separation between myofibers is a processing artifact. Treated fish show myodegeneration, with sarcoplasmic vacuolation in myofibers (arrows) and no obvious repair or inflammatory response. ***Testis***: The germinal epithelium is thin in contols and the seminiferous tubules contain abundant spermatozoa while testes from treated fish show degeneration with germ cell syncytia (thin blue arrow) and clusters of apoptotic germ cells having pyknotic nuclei and hypereosinophilic cytoplasm (yellow arrow). The germinal epithelium is thicker in treated fish than in controls with a higher proportion of spermatogonia and smaller lumina with fewer spermatozoa. ***Pancreas***: Acinar cells from controls contain fine red intracytoplasmic zymogen granules (arrow), but those from exposed fish are small due to granule depletion. ***Caudal kidney***: Sinusoidal capillaries are congested and distended with erythrocytes (yellow arrows) in treated fish.
