# Supplementary material for: Dichlorvos exposure results in large scale disruption of energy metabolism in the liver of the zebrafish, Danio rerio
Source: BMC Genomics. 2015 Oct 24;16:853. doi: 10.1186/s12864-015-1941-2 (PMC4619386; doi:10.1186/s12864-015-1941-2)
Supplement: Additional file 3: Table S1. — Functional analysis of differentially expressed genes. (PDF 2394 kb) [file 12864_2015_1941_MOESM3_ESM.pdf]

Table S1 Functional analysis of differentially expressed genes.

GOTM-KEGG pathway	Enrichment-Adjusted p-values			# of Probes Differentially Expressed			
	Low	Mid	High	Low (1056)	Mid (2654)	High (3497)	
Phenylalanine metabolism	3.2E-03	3.1E-01	~~deg~~	5	3	0	
Tyrosine metabolism	2.7E-03	3.1E-01	~~deg~~	6	4	0	
Phenylalanine, tyrosine and tryptophan biosynthesis	1.0E-03	2.4E-01	3.5E-01	4	2	3	
Selenoamino acid metabolism	1.0E-03	1.9E-01	~~deg~~	7	4	0	
Ubiquinone and other terpenoid-quinone biosynthesis	1.7E-02	2.8E-02	1.1E-01	3	3	4	
Tryptophan metabolism	1.0E-03	7.7E-05	4.7E-03	9	8	24	
Arginine and proline metabolism	1.5E-02	1.5E-02	1.2E-01	6	6	13	
Proteasome	~~deg~~	3.5E-06	6.9E-07	0	17	29	
Starch and sucrose metabolism	~~deg~~	1.6E-02	2.2E-02	0	4	13	Carbohydrate Metabolism
Pyruvate metabolism	3.0E-02	5.9E-03	1.1E-01	5	8	14	
Pentose phosphate pathway	1.5E-02	1.3E-02	4.2E-02	6	6	13	
Insulin signaling pathway	1.8E-02	6.2E-02	2.8E-01	13	13	31	
Glycolysis / Gluconeogenesis	5.2E-03	3.9E-02	2.7E-01	8	8	15	
Synthesis and degradation of ketone bodies	~~deg~~	2.8E-02	2.2E-02	0	3	8	Lipid Metabolism
Propanoate metabolism	9.3E-05	2.0E-04	2.2E-03	10	6	20	
Ether lipid metabolism	3.4E-01	2.8E-02	1.5E-01	3	7	13	
Glycerophospholipid metabolism	2.6E-01	2.8E-02	~~deg~~	5	6	0	
Glycerolipid metabolism	1.2E-02	1.3E-01	1.5E-01	6	6	14	
Biosynthesis of unsaturated fatty acids	2.5E-03	6.6E-03	4.7E-02	8	6	12	
Fatty acid elongation in mitochondria	2.3E-03	1.0E-02	4.7E-02	5	3	7	
PPAR signaling pathway	4.3E-02	9.0E-04	9.2E-02	7	13	21	
Butanoate metabolism	3.9E-03	1.0E-03	4.7E-03	8	7	18	
Fatty acid metabolism	3.4E-08	1.0E-08	1.0E-05	14	9	22	
Metabolism of xenobiotics by cytochrome P450	4.0E-01	2.5E-02	1.2E-01	3	5	13	Detoxification
Drug metabolism - other enzymes	3.1E-01	1.6E-02	5.2E-02	4	7	11	
Glutathione metabolism	1.0E-02	6.6E-03	6.9E-02	11	7	18	
Cysteine and methionine metabolism	1.5E-02	1.7E-01	3.5E-01	7	7	11	
Glycine, serine and threonine metabolism	4.5E-03	6.1E-03	6.7E-03	10	9	17	
Lysine degradation	3.2E-03	4.2E-03	1.8E-02	9	5	21	
β-Alanine metabolism	1.0E-03	6.6E-03	1.8E-02	7	3	12	
Valine, leucine and isoleucine degradation	4.0E-04	4.7E-03	4.7E-03	13	9	27	

Differentially espressed genes from all three exposure concentrations (Low, Mid, High) were submitted to to the KEGG pathway enrichment algorithms in GOTM to identify significantly enriched biological processes.The adjusted p-values of the enriched KEGG pathways and the number of probes in the pathway are shown. The total number of differentially expressed genes for each exposure is shown in parentheses in the column heading. The KEGG pathways were further clustered into the biofunctions shown at right. ~~deg~~ indicates that no differentially expressed genes were observed.
