# Supplementary material for: Dichlorvos exposure results in large scale disruption of energy metabolism in the liver of the zebrafish, Danio rerio
Source: BMC Genomics. 2015 Oct 24;16:853. doi: 10.1186/s12864-015-1941-2 (PMC4619386; doi:10.1186/s12864-015-1941-2)
Supplement: Additional file 4: Table S3. — Replication of microarray data using Quantigene assays. (PDF 1546 kb) [file 12864_2015_1941_MOESM4_ESM.pdf]

	Condition								
	DDVP high		Unfed 50 h		Unfed 29 h		Fed 4 h		
Gene symbol	Array	Quant	Array	Quant	Array	Quant	Array	Quant	Process / pathway
atg4b	5.7	6.8	-1.1	1.1	1.0	1.1	1.1	1.1	Autophagy
rab1a	1.9	2.5	1.1	1.5	1.0	1.4	1.0	1.1	Autophagy
echs1	-3.0	-2.1	1.0	1.2	1.0	1.6	1.2	1.1	Autophagy
hadhaa	-10.1	-9.3	-1.1	1.1	1.0	1.3	-1.1	1.2	Fatty acid synthesis
lpl	2.0	2.0	-1.1	-1.4	1.0	-1.2	-1.1	1.1	Fatty acid synthesis
calm3a	-2.2	-1.2	1.1	1.3	1.0	1.3	1.1	1.0	Glycogenolysis
g6pca	-2.9	-2.0	-1.9	1.0	1.0	1.2	-1.3	1.3	Glycolysis / Gluconeogenesis
ldhb	4.4	7.2	1.4	1.4	-1.1	1.5	1.4	1.3	Glycolysis / Gluconeogenesis
pgd	22.5	46.0	1.3	2.3	1.0	2.2	1.3	1.5	Pentose phosphate pathway
acat2	4.5	8.1	1.0	1.5	-1.1	2.2	2.8	1.8	Pyruvate metabolism
ephx1	-12.1	-10.2	1.0	1.3	1.0	1.5	1.1	1.1	Nrf2 canonical pathway (IPA)
gsr	31.8	52.1	1.6	1.6	1.0	1.0	-1.8	1.3	Nrf2 canonical pathway (IPA)
maff	10.7	16.7	1.0	1.7	1.0	1.0	-1.7	1.2	Nrf2 canonical pathway (IPA)

Table S3 Replication of selected microarray data using Quantigene gene expression assays. The geometric means of the fold-change differences from the control for each experiment (DDVP exposure and nutritional restriction, respectively; see Methods) are shown. We selected genes from several biological processes that were strongly affected by DDVP exposure but only minimally by nutritional deprivation in the microarray data sets. See **Additional file 6** (Table S3 Quantigene Probe Sequences) for probe details. Array: Microarray data. Quant: Quantigene data. Down-regulation is indicated by negative values.
