# Supplementary material for: Dichlorvos exposure results in large scale disruption of energy metabolism in the liver of the zebrafish, Danio rerio
Source: BMC Genomics. 2015 Oct 24;16:853. doi: 10.1186/s12864-015-1941-2 (PMC4619386; doi:10.1186/s12864-015-1941-2)
Supplement: Additional file 5: Table S4. — Gene, sequence and probe information for Quantigene probes. (PDF 1561 kb) [file 12864_2015_1941_MOESM5_ESM.pdf]

| Agilent Probe ID | Gene symbol | RefSeq ID | Quantigene Probe Sequence length | Quantigene Probe Probe set region | Process/pathway |
|---|---|---|---|---|---|
| A_15_P112958 | atg4b | NM_1089352 | 1799 | 501-1059 | Autophagy |
| A_15_P211031 | rab1a | NM_1007161 | 1820 | 155-728 | Autophagy |
| A_15_P119925 | echs1 | NM_1004529 | 1421 | 155-561 | Fatty acid synthesis |
| A_15_P161856 | hadhaa | NM_1105276 | 2785 | 1361-1759 | Fatty acid synthesis |
| A_15_P174191 | lpl | NM_131127 | 1904 | 727-1169 | Fatty acid synthesis |
| A_15_P115005 | calm3a | NM_182967 | 2180 | 48-548 | Glycogenolysis |
| A_15_P149541 | g6pca2 | NM_1163806 | 2331 | 9-504 | Glycolysis / Gluconeogenesis |
| A_15_P172771 | ldhba | NM_131247 | 1518 | 618-1039 | Glycolysis / Gluconeogenesis |
| A_15_P135551 | ephx1 | NM_201068 | 2121 | 809-1245 | Nrf2 canonical pathway (IPA) |
| A_15_P150356 | gsr | NM_1020554 | 2000 | 920-1426 | Nrf2 canonical pathway (IPA) |
| A_15_P578252 | maff | NM_200336 | 1485 | 213-619 | Nrf2 canonical pathway (IPA) |
| A_15_P184401 | pgd | NM_213552 | 1878 | 922-1312 | Pentose phosphate pathway |
| A_15_P101559 | acat2 | NM_131370 | 1954 | 537-987 | Pyruvate metabolism |
| A_15_P101632 | actb2 | NM_181601 | 1777 | 401-893 | Control/cytoskeleton |
| A_15_P213066 | rpl4 | NM_213107 | 1285 | 505-938 | Control/ribosomal protein |

**Table S4 Gene and sequence information for Quantigene probes.** Quantigene probes were designed by Affymetrix for genes of interest. The relevant Agilent microarray probe, and NCBI RefSeq identifiers are displayed. The length of the target sequence and region of the target sequence spanned by the Quantigene probes are also shown. See Table S2 for expression data.
