# Supplementary material for: Dichlorvos exposure results in large scale disruption of energy metabolism in the liver of the zebrafish, Danio rerio
Source: BMC Genomics. 2015 Oct 24;16:853. doi: 10.1186/s12864-015-1941-2 (PMC4619386; doi:10.1186/s12864-015-1941-2)
Supplement: Additional file 6: Table S2. — RefSeq IDs for Fig. 3a. (PDF 26 kb) [file 12864_2015_1941_MOESM6_ESM.pdf]

Table S2: Gene and sequence identifiers for Figure 3A.

Zebrafish Gene Symbol	Agilent Probe ID	Zebrafish RefSeq ID	Mouse Gene Symboll
pgd	A_15_P120264	NM_213453	PGD
taldo1	A_15_P107297	NM_199687	TALDO
pgm2l1	A_15_P195226	NM_001098485	PGM2L1
tktb	A_15_P116736	NM_198070	TKTL2
aldoc	A_15_P171261	NM_194384	ALDOC
dera	A_15_P149516	NM_001006097	DERA
g6pd	A_15_P183311	XM_694076	G6PD
gpia	A_15_P108859	NM_144763	GPI
gpib	A_15_P115695	NM_144764	GPI
rbks	A_15_P119149	NM_001002117	RBKS
fbp1	A_15_P140861	NM_213132	FBP1
txnrd1	A_15_P117668	NM_183072	TXNRD1
acaa1b	A_15_P111509	NM_001002207	ACAA1B
acox1	A_15_P104922	NM_001005933	ACOX1
hadha	A_15_P109636	NM_001089437	HADHA
elovl5	A_15_P101561	NM_200453	ELOVL5
elovl6	A_15_P108810	NM_199532	ELOVL6
hsd17b12	A_15_P109573	NM_200881	HSD17B12
oxsm	A_15_P182706	XM_687977	OXSM
cyb5r3	A_15_P110945	NM_212685	CYB5R3
ptplb	A_15_P114098	NM_199861	PTPLB
scd1	A_15_P209966	NM_198815	SCD1
acbd3	A_15_P113656	NM_213095	ACBD3
fth1	A_15_P116685	NM_131585	FTH1
gpx4a	A_15_P106339	NM_001007282	GPX4
gpx4b	A_15_P171266	NM_001030070	GPX4
gclc	A_15_P100082	NM_199277	GCLC
gss	A_15_P153431	NM_001006104	GSS
gsr	A_15_P107089	NM_001020554	GSR
prdx6	A_15_P118098	NM_200805	PRDX6
glrx5	A_15_P160621	NM_213021	GLRX5
glrx	A_15_P101071	NM_001002404	GLRX2
